# Identification of variants in *ACAN* and *PAPSS2* leading to spondyloepi(meta)physeal dysplasias in four Chinese families

**DOI:** 10.1002/mgg3.1916

**Published:** 2022-03-09

**Authors:** Yixuan Cao, Xin Guan, Shan Li, Nan Wu, Xiumin Chen, Tao Yang, Bo Yang, Xiuli Zhao

**Affiliations:** ^1^ Department of Medical Genetics, Institute of Basic Medical Sciences, Chinese Academy of Medical Sciences & School of Basic Medicine Peking Union Medical College Beijing China; ^2^ Department of Orthopedic Surgery, Peking Union Medical College Hospital Peking Union Medical College and Chinese Academy of Medical Sciences Beijing China

**Keywords:** bioinformatic analysis, short stature, spondyloepimetaphyseal dysplasia, spondyloepiphyseal dysplasia, variation, whole‐exome sequencing

## Abstract

**Background:**

Spondyloepi(meta)physeal dysplasias (SE[M]D) are a group of inherited skeletal disorders that mainly affect bone and cartilage, and next‐generation sequencing has aided the detection of genetic defects of such diseases. In this study, we aimed to identify causative variants in four Chinese families associated with SE(M)D.

**Methods:**

We recruited four unrelated Chinese families all displaying short stature and growth retardation. Clinical manifestations and X‐ray imaging were recorded for all patients. Candidate variants were identified by whole‐exome sequencing (WES) and verified by Sanger sequencing. Pathogenicity was assessed by conservation analysis, 3D protein modeling and in silico prediction, and was confirmed according to American College of Medical Genetics and Genomics.

**Results:**

Three novel SE(M)D‐related variants c.1090dupG, c.7168 T > G, and c.2947G > C in *ACAN*, and one reported variant c.712C > T in *PAPSS2* were identified. Among them, c.1090dupG in *ACAN* and c.712C > T in *PAPSS2* caused truncated protein and the other two variants led to amino acid alterations. Conservation analysis revealed sites of the two missense variants were highly conserved, and bioinformatic findings confirmed their pathogenicity. 3D modeling of mutant protein encoded by c.7168 T > G(p.Trp2390Gly) in *ACAN* proved the structural alteration in protein level.

**Conclusion:**

Our data suggested *ACAN* is a common pathogenic gene of SE(M)D. This study enriched the genetic background of skeletal dysplasias, and expanded the mutation spectra of *ACAN* and *PAPSS2*.

## Graphical abstract

- Pathogenic variants in *ACAN* and *PAPSS2* were identified in four Chinese families.
- Patients all presented short stature and were diagnosed with spondyloepi(meta)physeal dysplasias (SE(M)D).
- *ACAN* is a common pathogenic gene of SE(M)D.

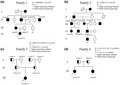

## 1 INTRODUCTION

Spondyloepi(meta)physeal dysplasias (SE(M)D) are a heterogeneous group of skeletal disorders with autosomal dominant/recessive or X‐linked inheritance. The main clinical manifestations include growth cessation, impaired development of vertebra, epiphysis, metaphysis, and advanced bone age, with the most prominent feature of short stature (Borochowitz et al., 2004; Cormier‐Daire, 2008; Dateki, 2017). Short stature is also the major reason for patients to seek for medical assistance. Based on clinical‐, radiological‐, and/or molecular phenotypes, SE(M)D can be classified into 35 subtypes, involving 34 different pathogenic genes (Table S1) (Mortier et al., 2019).

*ACAN* (MIM 155760) is one of the common pathogenic genes of SE(M)D and it encodes aggrecan, which is the most abundant non‐collagenous components in cartilage. The full‐length of *ACAN* gene contains 17 exons, and encodes 2530 amino acids. The aggrecan product consists of N‐terminal, G1 domain encoded by exons 2–5, IGD connecting region encoded by exon 6, G2 domain encoded by exons 7–9, keratin sulphate (KS) and chondroitin sulphate (CS) attachment region encoded by exons 10–11, G3 domain encoded by exons 12–17, and C‐terminal (Aspberg, 2012; Gibson & Briggs, 2016). The globular domain (G1‐G3) is the core protein region, and involves in mediating the interaction between various components in extracellular matrix. C‐type lectin repeat (CLD) regulates the binding of G3 domain to tenascin and fibulin, and KS‐CS acts as the attachment of chondroitin and keratan sulfate area, which produces highly negative charged molecules that hydrate cartilage tissue so that the cartilage can withstand high mechanical loading of the bones and joints (Aspberg, 2012). Homozygous variations in *ACAN* lead to SEMD, aggrecan type (SEMDAG) (MIM 612813, autosomal recessive), and heterozygous variation leads to SED, Kimberley type (SEDK) (MIM 608361, autosomal dominant) as well as short stature and advanced bone age, with or without early‐onset osteoarthritis and/or osteochondritis dissecans (MIM 165800, autosomal dominant). To date, two known mechanisms were proposed to cause *ACAN*‐related SE(M)D: premature termination codon‐induced mRNA degradation caused by nonsense/frameshift, and dominant‐negative effects caused by missense mutations (Briggs et al., 2015; Gibson & Briggs, 2016; Warman et al., 2011).

*PAPSS2* (MIM 603005) encodes PAPS (3′‐phosphoadenosine 5′‐phosphosulfate) synthase 2, which is widely involved in sulfidation of extracellular matrix proteins (Stelzer et al., 2007). The sulfation of extracellular matrix proteins plays a crucial role in development of chondrocytes (Schwartz & Domowicz, 2002), and defect in sulfate metabolism will lead to skeletal dysplasia. Spondyloepimetaphyseal dysplasia, Pakistani type (alternative name of Brachyolmia, MIM 612847, autosomal recessive), a distinct phenotype of SEMD, was first reported in 1998 (Ahmad et al., 1998). It was confirmed that *PAPSS2* was the disease‐causing gene and it follows autosomal recessive inheritance pattern (Iida et al., 2013; Miyake et al., 2012).

In this paper, we identified four variants in two cases of SEDK, one case of SEMDAG and one case of SEMD, Pakistani type, respectively. This study enriched the mutation spectra of *ACAN* and *PAPSS2*, and provided genetic basis for the precision molecular diagnosis of SE(M)D.

## 2 METHODS AND MATERIALS

### 2.1 Editorial policies and ethical considerations

This study was approved by Institutional Review Board (IRB) of the Institute of Basic Medical Sciences, Chinese Academy of Medical Sciences, Beijing, China (015‐2015). All participants or legal guardians of children under 18 signed their informed consent prior to their enrollment of this study.

### 2.2 Subjects

**FIGURE 1:**
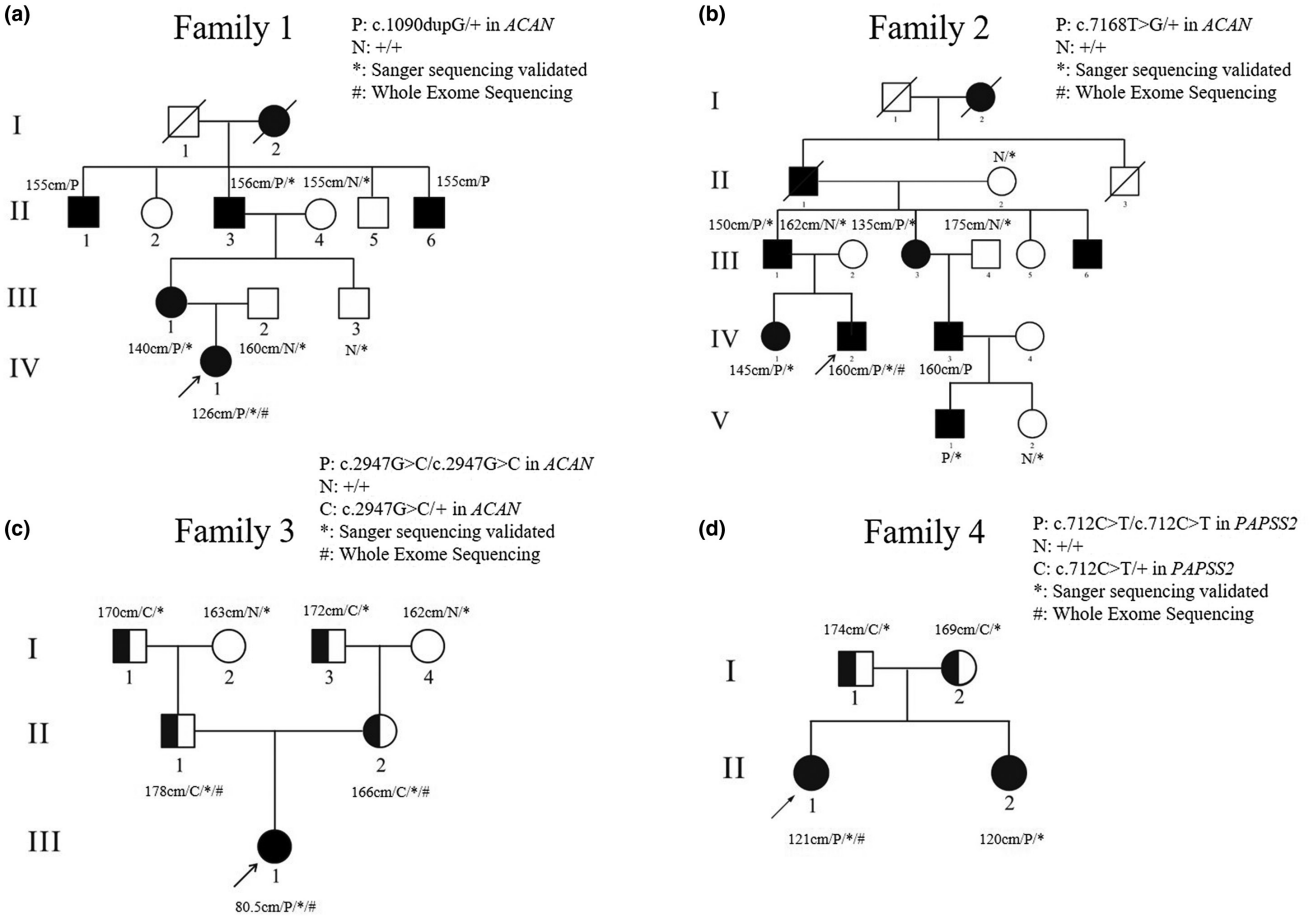
Pedigrees of four Chinese families with SE(M)D. Squares represent males and circles represent females. Affected members are in black symbols, and unaffected individuals are in open symbols. Symbols with half black indicate the carriers, and the arrows are pointed to the probands. Height (cm), genotypes, and whether WES or Sanger sequencing were performed were labeled next to the members with available data

Four SE(M)D Chinese families were enrolled in this study all presenting short stature and skeletal dysplasia (Figure 1). Peripheral blood samples of probands and available family members were collected, and clinical manifestations combined with imaging data and family history in the four families were recorded. Clinical data included age, gender, developmental history, height, weight, and presence of bone deformities. Height of children aged 0–7 was converted to age‐ and sex‐specific SDS z‐score based on data of Chinese population (Li, 2009). Height of adolescents over 7 years old was converted to age‐ and sex‐specific SDS z‐score according to corresponding provinces population data in China (Cai et al., 2012). For patients over 18 years old, *z*‐values were calculated based on the average height of population at aged 18.

### 2.3 Nomenclature

Genes in this study were named according to the HUGO Gene Nomenclature Committee (HGNC, https://www.genenames.org/), and all variants were named following the Human Genome Variation Society (HGVS) guideline (varnomen.hgvs.org). Sequences of cDNA, genomic DNA, and amino acids were obtained from University of California, Santa Cruz (UCSC) Genome Browser database (http://genome.ucsc.edu/), including genes *ACAN* (NM_13227, NG_012794.1) and *PAPSS2* (NM_001015880, NG_012150.1).

### 2.4 DNA isolation and whole‐exome sequencing (WES)

Genomic DNA was extracted from peripheral blood by traditional phenol chloroform method as describe previously (You et al., 2018). Hereafter, WES was performed on proband 1, proband 2, and proband 4 to detect the candidate disease causing variations. Trio WES was performed on proband 3 and her parents. Briefly, an Agilent liquid chip capture system was used to enrich the whole exon region of human DNA. Agilent Sure Select Human All Exon V6 kit was used to perform high‐throughput and high‐depth sequencing on a HiSeq 4000 (Illumina, San Diego, CA) platform. The average sequencing depth was 100×, and the detailed next‐generation sequencing process was described previously (Li et al., 2019).

### 2.5 Sanger sequencing

Sanger sequencing was performed for members with available blood samples (Family 1: II‐3, II‐4, III‐1, III‐2, III‐3, IV‐1; Family 2: II‐2, III‐1, III‐2, III‐3, III‐4, IV‐1, IV‐2, V‐1, V‐2; Family 3: I‐1, I‐2, I‐3, I‐4, II‐1, II‐2, III‐1; Family 4: I‐1, I‐2, II‐1, II‐2) to verify the candidate pathogenic variations identified by WES and further to confirm co‐segregation in the pedigrees. Primers were designed using online tool Primer 3 (https://bioinfo.ut.ee/primer3‐0.4.0/) (Table S2). Amplifications were performed using PCR system as follows: 95°C for 3 min; 94°C for 30 s, 58°C for 30 s, 72°C for 50 s (38 cycles); 72°C for 8 min. Amplicon was sequenced in Applied Biosystems 3730xl DNA Analyzer (Thermo Fisher Scientific, Waltham, MA, USA). Sequencing results were aligned to the reference sequences through CodonCode Aligner (version 6.0.2.6; CodonCode, Centerville, MA, USA). All variants identified in this study have been submitted to Leiden Open Variation Database (LOVD, http://www.lovd.nl), with IDs 00375198 (Family 1), 00375199 (Family 2), 00375200 (Family 3), and 00375201 (Family 4).

### 2.6 Bioinformatic analysis

Online Mendelian Inheritance of Man (OMIM; https://omim.org/) was employed to analyze the suspected pathogenic variants. UCSC was used to analyze the conservatism of variation sites, followed by sequence alignment using Molecular Evolutionary Genetics Analysis version X (MEGA‐X, https://www.megasoftware.net/) by comparing with different species. VarCards (http://varcards.biols.ac.cn/) was used to predict the effect of candidate variants by different algorithms. Pathogenicity was evaluated based on recommendation of American College of Medical Genetics and Genomics and the Association for Molecular Pathology (Richards et al., 2015). InterVar online database (http://wintervar.wglab.org/) was employed to evaluate the pathogenicity of variants. Swiss‐model (https://swissmodel.expasy.org/) was used to predict the protein structure of aggrecan by comparing the wild‐type and mutant proteins, followed by tertiary structure analysis using Swiss‐PdbViewer software (version 4.1.0). In this study, template used for ACAN protein modeling was based on aggrecan core protein (PBD: 1tdq) with the highest GMQE score for the protein tertiary structure analysis.

## 3 RESULTS

### 3.1 Clinical manifestations

In this study, we recruited four unrelated Chinese families all presenting short stature and skeletal dysplasias of long bones and spine. The detailed clinical manifestations of patients are shown in Table 1.

**TABLE 1:** Clinical manifestations recorded in four probands associated with SE(M)D

Proband	I	II	III	IV
Sex	F	M	F	F
Age at visit (years)	31	14	2	26
Birth height (cm)	NA	NA	48	53
Height at visit (cm/SDS z‐score)	126/−7 SD	160/−1 SD	80.5/−2 SD	121/−7 SD
Birth weight (kg)	NA	4	3.33	3.9
Current weight (kg)	35	75	10.5	41
Parental height (Father cm/Mother cm)	160/140	150/162	178/166	174/169
Large head circumference	−	−	NA	−
Coarse facies	−	−	−	−
Brachydactyly	+	+	+	+
short limbs	+	−	+	+
Leg deformity	+	+	−	+
Joint injury or arthritis	−	+	−	−
Epiphyseal dysplasia	NA	+	+	NA
Pectus carinatum	+	+	−	−
Kyphosis	−	−	−	+
Abnormal ribs	NA	−	−	NA
Fleshy prominent heels	NA	NA	−	−

*Notes*: NA, not available; +, the feature is present; −, the feature is not present.

**FIGURE 2:**
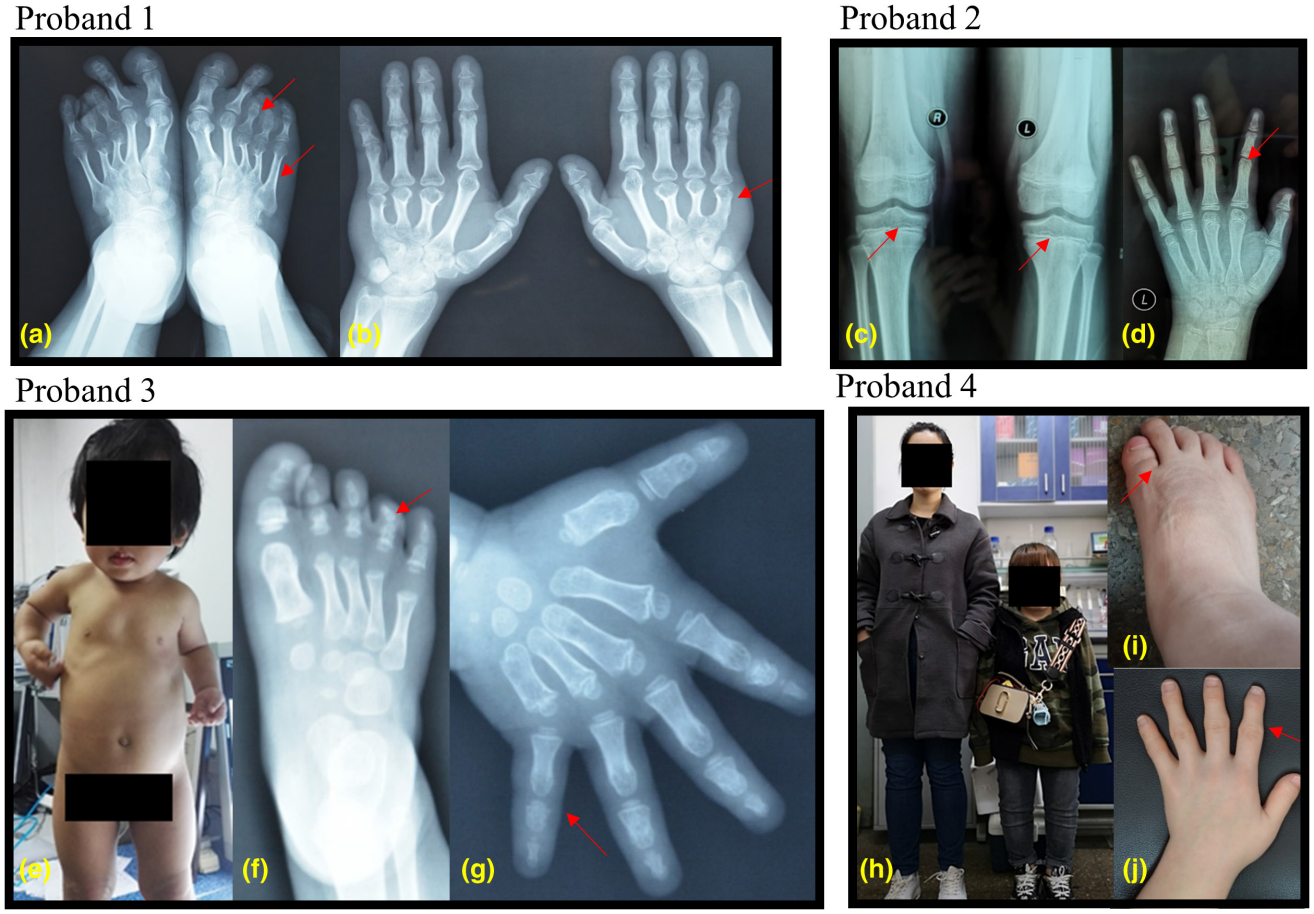
Clinical characteristics of the four probands. (a and b) Metatarsal dysplasia, phalangeal dysplasia, and metacarpal dysplasia in proband 1; (c and d) Decreased bone density in epiphyses and underdevelopment of first distal phalanx in hand in proband 2. (e–g) Short and disproportionate upper limbs (e), and blurring growth plate region in proband 3. (h–j) Short stature (h), abnormal trochlea phalanges (i), and short middle/distal phalanx (j) in proband 4

Proband 1 in pedigree 1 had short stature (−7 SD), with metatarsal dysplasia, phalangeal dysplasia, metacarpal dysplasia (Figure 2a,b), and genu varus legs. Proband 1 had a family history, and the phenotype of other affected families were similar to proband 1. Proband 2 in pedigree 2 presented mild short stature (−1 SD), pectus carinatum, joint pain, and displayed symptoms of early‐onset osteoarthritis. The X‐ray images revealed slightly widened metaphysis of bilateral femur and tibia, with decreased bone density in epiphyses (Figure 2c). Decreased bone density was also found in bilateral hands with thin metacarpal and phalangeal cortex, and underdevelopment of bilateral first distal phalanx in hand was noticed as well (Figure 2d). Some patients carrying the same variants in family 2 developed severe bone deformation, and the male patients displayed much severe phenotype than female patients. Proband 3 in pedigree 3 had a height of −2 SD, presenting growth retardation and short limbs (Figure 2e). Radiographic examinations revealed a narrow growth plate region, and presented joint epiphysis dysplasia (Figure 2f,g). Proband 4 in pedigree 4 was born to non‐consanguineous parents. She presented growth retardation with a height of −7 SD (Figure 2h), and displayed enlarged joints, joint closure, and growth arrest after 12 years old. Brachydactyly with abnormal trochlea phalanges (Figure 2i), short middle/distal phalanx (Figure 2j), and kyphosis of thoracic spine were found in the patient.

### 3.2 Identification of variants

**FIGURE 3:**
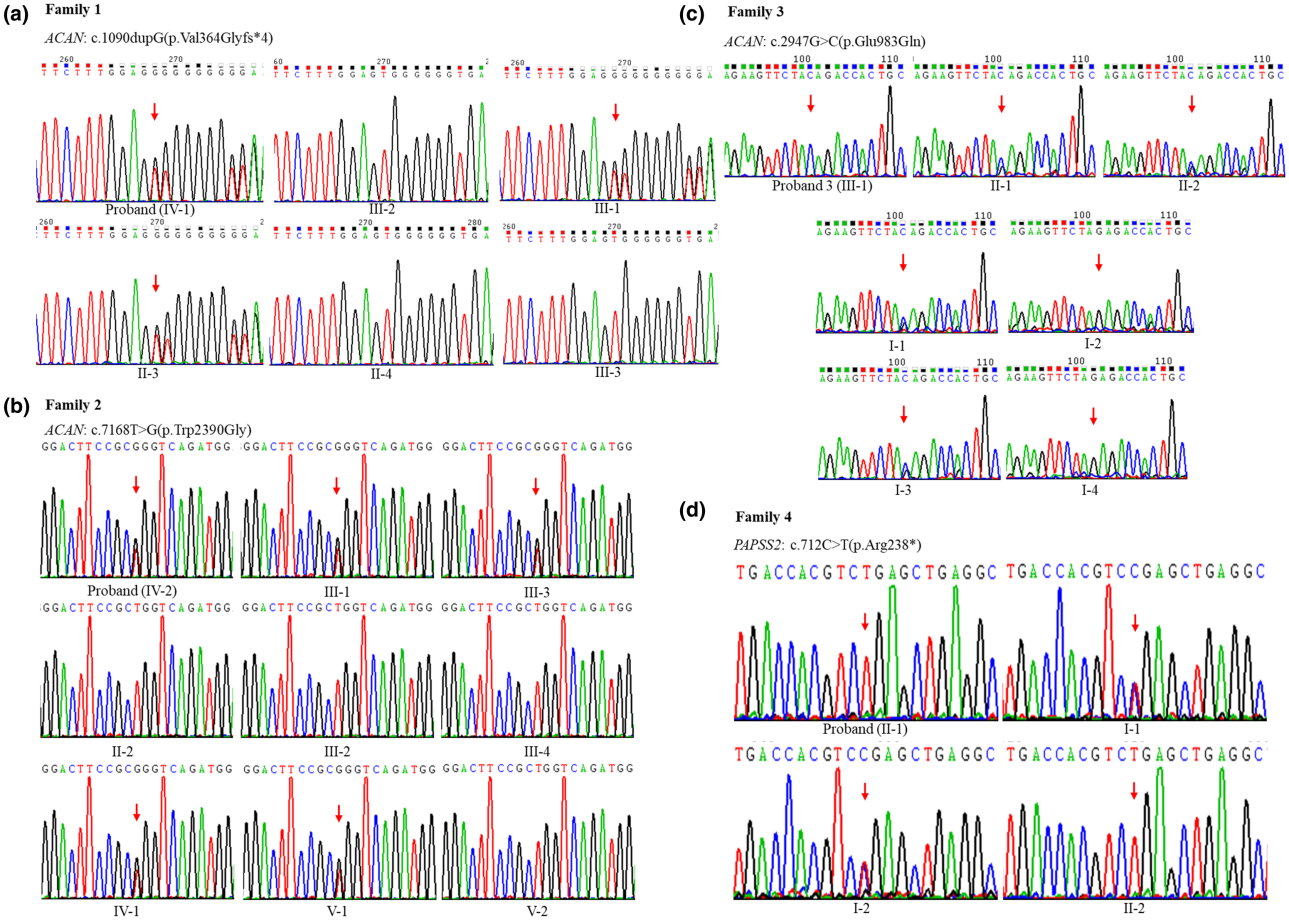
Sequencing analysis of four variants associated with SE(M)D. Sequencing analysis of all available members in families 1–4. (a) In family 1, II‐3, III‐1, and IV‐1 were heterozygous of c.1090dupG(p.Val364Glyfs*4) in *ACAN*. (b) In family 2, III‐1, III‐3, IV‐1, IV‐2, and V‐1 were heterozygous of c.7168 T > G(p.Trp2390Gly) in *ACAN*. (c) In family 3, III‐1 was homozygous of c.2947G > C(p.Glu983Gln) in *ACAN*, while parents II‐1, II‐2, and grandfathers I‐1, I‐3 were heterozygous of the variant. (d) In family 4, II‐1 and II‐2 were homozygous of c.712C > T(p.Arg238*) in *PAPSS2*, and the parents (I‐1, I‐2) were heterozygous of the variant. Red arrows point to the mutation sites

Four pathogenic variants related to SE(M)D were detected by WES combined with Sanger sequencing (Figure 3). Variants in *ACAN* were detected in families 1–3 (Table 2). Heterozygous variant c.1090dupG in *ACAN* was identified in proband 1, which caused frameshift and generated a premature termination codon (p.Val364Glyfs*4). A heterozygous missense variant c.7168 T > G in *ACAN* was identified in proband 2, which led to a substitution of tryptophan to glycine (p.Trp2390Gly). Homozygous variant c.2947G > C(p.Glu983Gln) in *ACAN* was detected in proband 3. Based on the genotype and clinical manifestations, probands 1 and 2 were diagnosed as SEDK, and proband 3 was diagnosed as SEMDAG. Homozygous variants c.712C > T(p.Arg238*) in *PAPSS2* were identified in the proband 4 and her younger sister. Both of their parents were the carriers of variant c.712C > T. Based on the clinical manifestations and genetic variations, proband 4 was diagnosed as SEMD, Pakistani Type. The above four pedigrees were verified to be in accordance with the family co‐segregation (Figure 3).

**TABLE 2:** Identification and bioinformatic analysis of causative variants in four families

	Family 1	Family 2	Family 3	Family 4
Pathogenic genes	*ACAN*	*ACAN*	*ACAN*	*PAPSS2*
Transcript	NM_13227	NM_13227	NM_13227	NM_001015880
Nucleotide change	c.1090dupG	c.7168 T > G	c.2947G > C	c.712C > T
Protein change	p.Val364Glyfs*4	p.Trp2390Gly	p.Glu983Gln	p.Arg238*
Family inheritance pattern	Autosomal dominant	Autosomal dominant	Autosomal recessive	Autosomal recessive
Frequency (gnomAD_exome_All)	NA	NA	0.000100	0.000016
MAF (gnomAD_East Asian_Exomes)	NA	NA	0.001468	0.000000
Novelty (HGMD Professional, release 2021.04)	Novel	Novel	Novel	Reported
Disease classification	Spondyloepiphyseal dysplasia, Kimberley type	Spondyloepiphyseal dysplasia, Kimberley type	Spondyloepimetaphyseal dysplasia, Aggrecan type	Spondyloepimetaphyseal dysplasia, Pakistani type
SIFT	NA	0.0(Da)	0.035(Da)	NA
Polyphen‐2	NA	0.957(PD)	0.985(PD)	NA
LRT	NA	0.009(N)	NA	0.009(N)
MutationTaster	1(DC)	0.99(DC)	1(P)	1(DC)
MutationAssessor	NA	3.995(H)	2.275(M)	NA
FATHMM	NA	1.2(T)	−3.9(Da)	NA
PROVEAN	NA	−10.37(Da)	−0.95(N)	NA
VEST3	NA	0.893(Da)	0.16(T)	NA
M‐CAP	NA	0.061(Da)	0.105(Da)	NA
CADD	NA	32(Da)	19.69(T)	40(Da)
DANN	NA	0.980(T)	0.996(Da)	0.998(Da)
FATHMM_MKL	NA	0.965(Da)	0.219(T)	0.500(T)
Eigen	NA	0.950(Da)	−0.014(T)	0.440(Da)
GenoCanyon	NA	1.000(Da)	0.010(T)	0.984(T)
fitCons	NA	0.487(T)	0.516(T)	0.722(Da)
ACMG Assessment	PVS1 PM2 PP1	PM1 PM2 PP1 PP3	BS1 PP3	PVS1 PM2 PP3 PP5
ACMG Classification	PG	LP	US	PG

Abbreviations: B, benign; Da, damaging; DC, disease causing; De, deleterious; H, high; LP, likely pathogenic; M, medium; N, neutral; NA, not available; P, polymorphism; PD, probably damaging; PG, pathogenic; T, tolerable; US, uncertain significance.

### 3.3 Pathogenicity analysis

**FIGURE 4:**
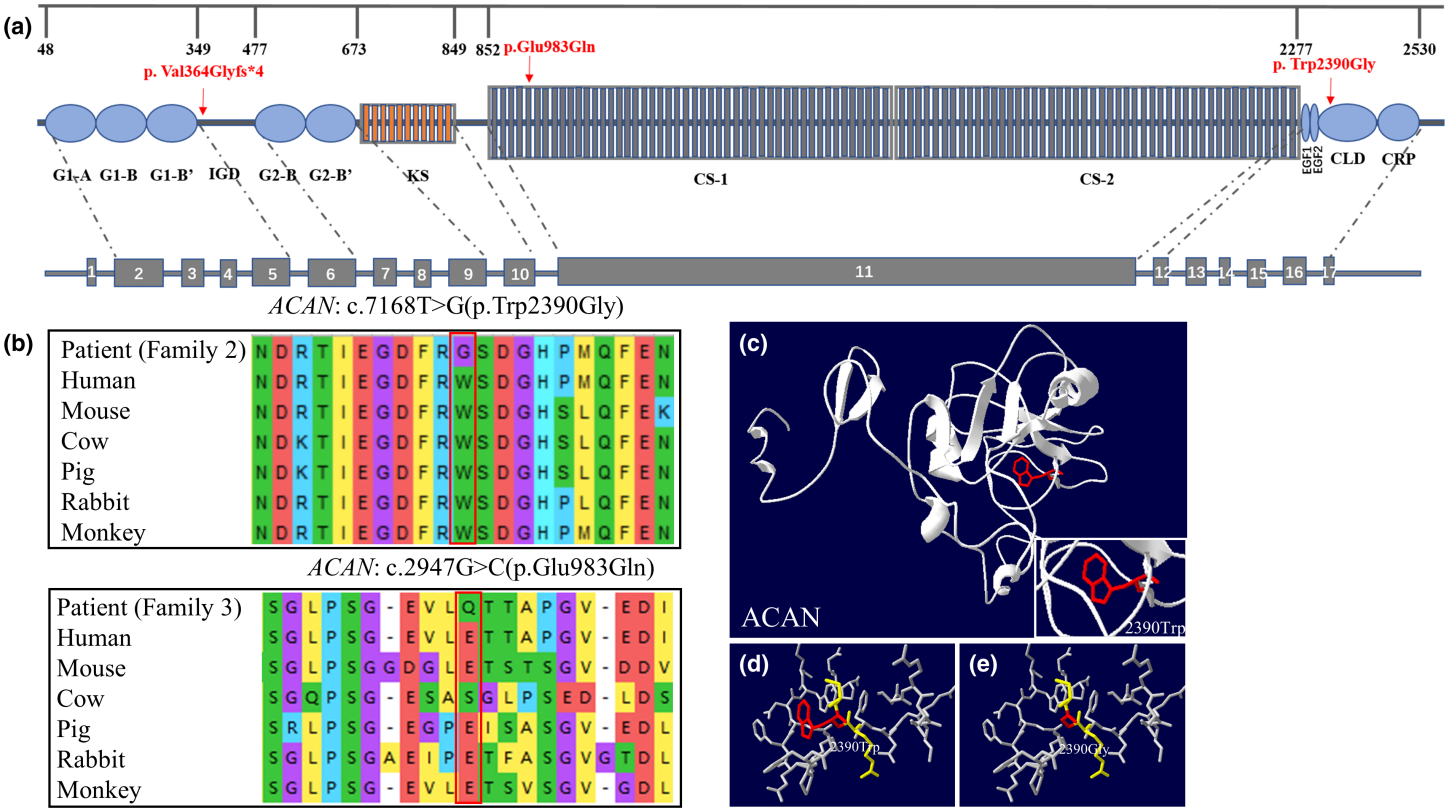
Bioinformatic prediction by conservation analysis and 3D protein structure. (a) Structure of aggrecan containing the main domains that are drawn to scale is shown above. Structure of the *ACAN* is shown below, and the numbers indicate the corresponding exons. Variants reported in this study are labeled in red. G, globular domain; IGD, interglobular domain; KS, keratan sulfate attachment region; CS, chondroitin sulfate attachment region; EGF, epidermal growth factor‐like domain; CLD, C‐type lectin domain; CRP, complement regulatory‐like domain. (b) Sequence alignment of two missense variants in *ACAN* was analyzed by comparing the amino acid sequences in human, mouse, cow, pig, rabbit, and monkey. (c–e) Overall structure of the ACAN protein (c), tertiary structure of the wild‐type (d), and p.Trp2390Gly mutant protein (e)

To analyze the conservatism of variant sites, the missense variants, c.7168 T > G(p.Trp2390Gly), and c.2947G > C(p.Glu983Gln) in *ACAN* were subjected to conservation analysis by performing multiple sequence alignment among different species. The results showed both of these mutant sites were highly conserved (Figure 4b). The pathogenicity of the variants was predicted through VarCards (Table 2), based on different in silico tools. In accordance with ACMG classification (Richards et al., 2015), the variants in Family 1–4 were classified as pathogenic, likely pathogenic, uncertain significance, and pathogenic, respectively (Table 2).

Protein structure was imitated using Swiss‐model. Up to now, there is no fully resolved model of the whole aggrecan protein, so the missense variant c.2947G > C(p.Glu983Gln) of proband 3 cannot be predicted through this method. By analyzing the protein structure, we found that the variant c.7168 T > G(p.Trp2390Gly) in *ACAN* (Figure 4c–e) changed tryptophan to glycine at the mutant site, but did not change the structure of hydrogen bond. However, alteration from nonpolar tryptophan to polar glycine may affect the charge distribution on the protein surface.

## 4 DISCUSSION

In this study, we investigated four unrelated Chinese families associated with SE(M)D caused by monogenic defect, and four variants were identified in *ACAN* and *PAPSS2*.

Among the four variants, three of them located in *ACAN*, including p.Val364Glyfs*4, p.Trp2390Gly, and p.Glu983Gln, and they located at IGD, CLD, and CS attachment domain of *ACAN*, respectively (Figure 4a). Studies have shown that the globular domain G1‐G3 mediates the interaction between various components of extracellular matrix, missense variant in CLD domain may affect the development of cartilage and growth plate, and KS‐CS domain regulates the high mechanical load capacity of cartilage (Domowicz et al., 2009). It is speculated that the variants in key structural domains affect the function of aggrecan and thus interrupt the development and formation of cartilage (Lauing et al., 2014). Our data showed missense variants p.Trp2390Gly and p.Glu983Gln were highly conserved in different species and were confirmed to be pathogenic under the assessment of at least five different bioinformatic methods such as SIFT and polyphen‐2 (Figure 4b; Table 2). Although the variant p.Trp2390Gly did not affect the tertiary structure hydrogen bond of aggrecan, the transformation from non‐polar tryptophan to polar glycine may affect the charge distribution on the protein surface (Figure 4c–e).

Aggrecan, the protein coded by *ACAN*, is a member of lectican family and is predominant proteoglycan in cartilage extracellular matrix (Tompson et al., 2009). The pathogenesis of SE(M)D is due to the disrupted interaction with tenascins and other extracellular matrix proteins induced by the mutations in *ACAN* (Tompson et al., 2009). Until now, there are two proposed mechanisms of *ACAN*‐induced SE(M)D being either haploinsufficiency or dominant‐negative effect (Gibson & Briggs, 2016). Haploinsufficiency is generally resulted from premature termination codons through nonsense‐mediated mRNA degradation. The variant c.1090dupG(p.Val364Glyfs*4) in *ACAN* in Family 1 was therefore presumed haploinsufficiency for aggrecan. Dominant‐negative effect caused by missense mutations will mostly affect cartilage structure and function by losing fibulin‐1/2 and tenascin‐R interactions (Gibson & Briggs, 2016; Stattin et al., 2010). Patients in family 2 with Trp2390Gly located at C type lectin domain, possibly developed SEDK following this mechanism. While patient in family 3 with homozygous Glu983Gln, its etiology is likely also due to dominant‐negative effect, however a full understanding of how this is contributed to SEMDAG remains to be elucidated.

Regarding genotype–phenotype relationship among variants in *ACAN*, it is believed that there is no remarkable correlation based on previous studies (Gkourogianni et al., 2017; Hauer et al., 2017). Nevertheless, it has been shown that patients with heterozygous *ACAN* variants displayed a milder phenotype (Gkourogianni et al., 2017; Sentchordi‐Montane et al., 2018), with brachydactyly, short stature, and milder skeletal dysplasia (Sentchordi‐Montane et al., 2018). Hu et al. showed a concentration of null variants located at 5′ of *ACAN* and all of them contributed to non‐syndromic short stature with no signs of skeletal abnormities (Hu et al., 2017), indicating null variants at 5′ end of *ACAN* mainly affect growth plate. While the 3′ end of *ACAN*, with variants located at CLD, potentially affect both articular and growth plate cartilage (Hu et al., 2017; Nilsson et al., 2014). It is known that early‐onset osteoarthritis (OA) is a main clinical manifestation in SE(M)D patients (Gkourogianni et al., 2017). However, in our study only proband 2 with variants at CLD showed OA, which was consistent with the previous report by Nilsson et al. and further provided evidence of the crucial role of CLD in cartilage development (Gkourogianni et al., 2017; Sentchordi‐Montane et al., 2018).

Until now, heterozygous *ACAN* variants have been reported in approximately 40 families worldwide and only two reports of SEMDAG caused by homozygous *ACAN* variants (Fukuhara et al., 2019; Stavber et al., 2020; Tompson et al., 2009). Tompson et al. (2009) firstly reported SEMDAG patients with extreme short stature, short necks, barrel chests, brachydactyly, craniofacial abnormalities, lumbar lordosis, telescoping interphalangeal joints, and enlarged long bone metaphysis. Fukuhara et al. (2019) reported the second case with a milder phenotype, who showed short stature, cervical lordosis, thoracolumbar kyphosis, and hip joint degenerative lesions. Here, we reported the third case (pedigree 3), presenting short stature, short limbs, and abnormal joint epiphyses with extremities, but no severe facial dysplasia (Figure 2e–g), which was similar to the second case. In addition, we noticed foot varus, muscle weakness, and inability to bounce in our SEMDAG patient. Our report further enriched the clinical manifestations of SEMDAG, however the correlation between genotypes and phenotypes in SEMDAG patients need to be clarified with larger population.

We identified a previously reported (Stranneheim et al., 2021) homozygous variants c.712C > T(p.Arg238*) in *PAPSS2*. As a synthase, the protein encoded by *PAPSS2* participates in the sulfation process of extracellular matrix proteins, and thereby plays an important role in the proliferation and differentiation of chondrocytes (Schwartz & Domowicz, 2002). It was shown that *PAPSS2* provides sulfate donor and responsible for sulfation of dehydroepiandrosterone (DHEA) (Oostdijk et al., 2015). Till now, *PAPSS2* was known to cause SEMD, Pakistani type/Brachyolmia with main clinical characteristics of disproportionate short stature, short spine, kyphosis, scoliosis, enlarged knee joints, bowed legs, irregular epiphyses, narrow carpal space, and mild brachydactyly (Ahmad et al., 1998; Bownass et al., 2019; Miyake et al., 2012; Noordam et al., 2009; Tuysuz et al., 2013). The patient with *PAPSS2* variants in our study showed typical clinical manifestations including short stature, brachydactyly, kyphosis, and knee swelling, which were similar to the phenotypes in previous reports. Interestingly, patient with *PAPSS2* variants showed kyphosis (Table 1) which was absent in patients with *ACAN* variants, indicating a distinct and important role of *PAPSS2* in vertebral development. Though there are different types of variants causing SEMD, Pakistani type, loss‐of‐function variants are predominant (Miyake et al., 2012), which is also the case in our study. Miyake et al. showed that patients with loss‐of‐function variants in *PAPSS2* all displayed similar spinal changes, however with variable epiphyseal and metaphyseal phenotypes (Miyake et al., 2012). The proband 4 with loss‐of‐function variants in *PAPSS2* in our study also showed spinal dysplasia however, the data of epiphyseal/ metaphyseal were not available.

In summary, we identified four variants in *ACAN* and *PAPSS2* from four Chinese families associated with SE(M)D. This study expands the genotypic–phenotypic spectra of *ACAN* and *PAPSS2*, and highlights the important role of *ACAN* in short stature‐related skeletal dysplasia.

## CONFLICT OF INTEREST

The authors declare that the research was conducted in the absence of any commercial or financial relationships that could be construed as a potential conflict of interest.

## AUTHOR CONTRIBUTIONS

*Performed the experiment and prepared the manuscript*: Yixuan Cao and Xin Guan. *Designed the primers, and contributed to part of experiment*: Shan Li. *Responsible for recording of clinical data*: Xiumin Chen and Tao Yang. *Contributed to the patients' samples collection*: Nan Wu. *Designed and supervised this research*: Xiuli Zhao and Bo Yang. All authors performed critical reading and approved the final version of manuscript.

## ETHICAL STATEMENT

This study was approved by Institutional Review Board (IRB) of the Institute of Basic Medical Sciences, Chinese Academy of Medical Sciences, Beijing, China (015‐2015). Written informed consent was obtained from all participants/legal guardians of children under 18. All methods were performed in accordance with the guidelines of the Declaration of Helsinki.

## Supporting information


Tables S1 and S2

## Data Availability

The data that support the findings of this study are available on request from the corresponding author.
