# Supplementary material for: Identification of variants in ACAN and PAPSS2 leading to spondyloepi(meta)physeal dysplasias in four Chinese families
Source: Mol Genet Genomic Med. 2022 Mar 9;10(5):e1916. doi: 10.1002/mgg3.1916 (PMC9034684; doi:10.1002/mgg3.1916)
Supplement: Supplementary file 1 — Tables S1 and S2 [file MGG3-10-e1916-s001.docx]

**Supplementary Materials**

**Table S1. Classification of SE(M)D**

| **Name of disorder** | **Inheritance** | **Pathogenic gene** | **OMIM number** |
| --- | --- | --- | --- |
| Spondyloepiphyseal dysplasia congenita (SEDC) | AD  AR | *COL2A1* | 183900  616583  604864 |
| Spondyloepiphyseal dysplasia with marked metaphyseal changes (SEMD) | AD | *COL2A1*  *FN1* | 184250  184255 |
| SED with metatarsal shortening (formerly Czech dysplasia) | AD | *COL2A1* | 609162 |
| SEMD, PAPSS2 type | AR | *PAPSS2* | 612847 |
| SED, Kimberley type | AD | *ACAN* | 608361 |
| SEMD, Aggrecan type | AR | *ACAN* | 612813 |
| Spondyloepimetaphyseal dysplasia, Maroteauxtype (pseudo-Morquio syndrome type 2) | AD | *TRPV4* | 184095 |
| Spondylometaphyseal dysplasia, Sedaghatian type | AR | *GPX4* | 250220 |
| Severe spondylometaphyseal dysplasia (SMD Sedaghatian-like) | AR | *SBDS* |  |
| SED, MIR140 type | AD | *MIR140* | 618618 |
| SEMD with joint laxity (SEMD-JL), leptodactylic or Hall type | AD | *KIF22* | 603546 |
| SEMD with joint laxity (SEMD-JL), Beighton type | AR | *B3GALT6* | 271640 |
| SEMD with joint laxity (SEMD-JL), EXOC6B type | AR | *EXOC6B* | 618395 |
| Dyggve–Melchior–Clausen dysplasia (DMC) | AR  AR | *DYM*  *RAB33B* | 223800  615222 |
| Immuno-osseous dysplasia (Schimke) | AR | *SMARCAL1* | 242900 |
| SED with diabetes mellitus, Wolcott–Rallison  type | AR | *EIF2AK3* | 226980 |
| SEMD, Matrilin type | AR | *MATN3* | 608728 |
| SEMD, Shohat type | AR | *DDRGK1* | 602557 |
| SEMD with leukodystrophy, AIFM1 type | XL | *AIFM1* | 300232 |
| SEMD, biglycan type | XL | *BGN* | 300106 |
| SEMD with immune deficiency, EXTL3 type | AR | *EXTL3* | 617425 |
| SEMD with intellectual disability, NANS type | AR | *NANS* | 610442 |
| SEMD with intellectual disability, RSPRY1 type | AR | *RSPRY1* | 616723 |
| SEMD, TMEM165 type | AR | *TMEM165* | 614727 |
| SEMD, PISD type | AR | *PISD* |  |
| SEMD, UFSP2 type | AD | *UFSP2* | 617974  142669 |
| SEMD, short limb–abnormal calcification type | AR | *DDR2* | 271665 |
| SED tarda, X-linked (SED-XL) | XL | *TRAPPC2* | 313400 |
| Ehlers–Danlos syndrome, spondylodysplastic  type | AR | *SLC39A13* | 612350 |
| SPONASTRIME dysplasia | AR | *TONSL* | 271510 |
| Platyspondyly (brachyolmia) with amelogenesis  imperfecta | AR | *LTBP3* | 601216 |
| CODAS syndrome | AR | *LONP1* | 600373 |
| EVEN-PLUS syndrome | AR | *HSPA9* | 616854 |
| CAGSSS syndrome | AR | *IARS2* | 616007 |
| Steel syndrome | AR | *COL27A1* | 615155 |

**Table S2. Primers designed for PCR**

| **Gene name** | **Family number** | **Primer name** | **Sequence(5’→3’)** | **Tm(℃)** | **Product size（bp）** |
| --- | --- | --- | --- | --- | --- |
| *ACAN* | 1 | E6-F | CCGTCAGCTCATTGGTCAAT | 58 | 718 |
|  |  | E6-R | AGCCTTCAACTGCGGGAATC |  |  |
|  | 2 | E14-F | CCAGAGTCAGCCCAAGACTA | 58 | 624 |
|  |  | E14-R | GTTCAGTGTGGGCAAGAAAC |  |  |
|  | 3 | E11-F | GAGATCCTAGAGGGCTCTGC | 58 | 1880 |
|  |  | E11-R | CCACTGAGGTCAGTCCCTAC |  |  |
| *PAPSS2* | 4 | E6-F | AGATGCGATGATTGTCACCC | 58 | 577 |
|  |  | E6-R | CCTTGTGGCAATTCTTGGTG |  |  |
